# Structure and Innervation of the Extrahepatic Biliary System in the Australian Possum, Trichosurus Vulpecula

**DOI:** 10.1155/1993/72946

**Published:** 1993

**Authors:** R. T. A. Padbury, R. A. Baker, J. P. Messenger, J. Toouli, J. B. Furness

**Affiliations:** 1 Department of Surgery Flinders Medical Centre Bedford Park SA 5042 Australia; 2 Department of Anatomy and Cell Biology University of Melbourne Parkville VIC 3052 Australia

## Abstract

The morphology, microanatomy and innervation of the biliary tree of the Australian possum, Trichosurus vulpecula, was examined. The gross morphology of the gallbladder, hepatic and cystic ducts, and the course of the common bile duct, conforms to those of other species. The sphincter of Oddi has an extraduodenal segment that extends 15mm from the duodenal wall; within this segment the pancreatic and common bile ducts are ensheathed together by sphincter muscle. Their lumens unite to form a common channel within the terminal intraduodenal segment.

Nerve cell bodies of the gallbladder were found in an inter-connecting network of ganglia that were located in the serosa, muscularis and mucosa. Nerve fibres innervated the muscle, arterioles and the mucosa. Few ganglia were found along the supra sphincteric portion of the common bile duct. Nerve trunks followed the duct and a dense nerve fibre plexus was found in the mucosa. In the sphincter most ganglia were located in two plexuses, the first between the layers of the external sphincter muscle, which was continuous with the external muscle of the duodenum, and the second was associated with the internal sphincter muscle. Nerve fibres were numerous in the sphincter muscle, and were also found in the subepithelial and periglandular plexuses of both the pancreatic and common bile ducts.


*HPB Surgery*, 1993, Vol. 7, pp. 125–140
Reprints available directly from the publisher
Photocopying permitted by license only

© 1993 Harwood Academic Publishers GmbH
Printed in the United States of America

### STRUCTURE AND INNERVATION OF THE EXTRAHEPATIC BILIARY SYSTEM IN THE AUSTRALIAN POSSUM, TRICHOSURUS VULPECULA

R.T.A PADBURY[1], R.A. BAKER[1], J.P. MESSENGER[2], J. TOOULI[1] and J.B. FURNESS[2]

[1]*Department of Surgery, Flinders Medical Centre, Bedford Park, SA 5042, Australia*

[2]*Department of Anatomy and Cell Biology, University of Melbourne, Parkville, VIC 3052, Australia*

*(Received 27 January 1993)*

The morphology, microanatomy and innervation of the biliary tree of the Australian possum, Trichosurus vulpecula, was examined. The gross morphology of the gallbladder, hepatic and cystic ducts, and the course of the common bile duct, conforms to those of other species. The sphincter of Oddi has an extraduodenal segment that extends 15mm from the duodenal wall; within this segment the pancreatic and common bile ducts are ensheathed together by sphincter muscle. Their lumens unite to form a common channel within the terminal intraduodenal segment.

Nerve cell bodies of the gallbladder were found in an inter-connecting network of ganglia that were located in the serosa, muscularis and mucosa. Nerve fibres innervated the muscle, arterioles and the mucosa. Few ganglia were found along the supra sphincteric portion of the common bile duct. Nerve trunks followed the duct and a dense nerve fibre plexus was found in the mucosa. In the sphincter most ganglia were located in two plexuses, the first between the layers of the external sphincter muscle, which was continuous with the external muscle of the duodenum, and the second was associated with the internal sphincter muscle. Nerve fibres were numerous in the sphincter muscle, and were also found in the subepithelial and periglandular plexuses of both the pancreatic and common bile ducts.

KEY WORDS: Biliary tract, Sphincter of Oddi, Australian possum, Trichosurus vulpecula, enteric nervous system

### INTRODUCTION

The neural architecture of the biliary tract has not been comprehensively examined in a single species. Most studies have concentrated on the gallbladder, but a consistent description of the arrangement of neural plexuses has not yet emerged.

The Australian brush-tailed possum, Trichosurus vulpecula, is an appropriate species in which to examine the microanatomy and innervation of the biliary system

Reprint requests to: Dr R.T.A. Padbury, Department of Surgery, Flinders Medical Centre, Bedford Park, SA 5042, Australia because of its suitability for physiological studies[1,2,3]. Like the American opossum, this species has an extraduodenal sphincter of Oddi, which greatly simplifies studies of sphincter function. However, knowledge of the innervation of the biliary system, which would enable correlated structural and functional studies, is lacking.

Examination of immunoreactivity for neuron specific enolase (NSE) and the glial protein, S100, both of which can be used to study the complete neural network, were systematically applied to map the neural architecture of the biliary tract. Comparisons were made with other studies in different species, and the relationship between the enteric nervous plexuses of the intestine and those of the biliary tract examined.

### MATERIALS AND METHODS

Adult possums of either sex weighing 700–2100g were used. All possums were kept in individual cages. Animal sacrifice was by intraperitoneal injection of 200–300mg of sodium pentobarbitone (Nembutal, Bomac Laboratories, NSW, Australia). The extrahepatic biliary system and duodenum were dissected in three fasted animals; the anatomic relationships were recorded and the gross dimensions of its components measured.

Specimens to be examined histologically were removed and washed in sodium phosphate buffered sodium chloride (PBS; 0.01M sodium phosphate buffer, 0.9% NaCl, pH 7.0) and any particulate matter or intestinal content was removed. The specimens were then pinned flat on balsa wood and fixed in 2% formaldehyde in 0.1M sodium phosphate buffer plus 15% of a saturated solution of picric acid (final pH 7.0) at 4° C. After overnight fixation the specimens were dehydrated in dimethylsulphoxide (3 changes of 10 min each) and then rehydrated in PBS (3 further changes of 10 min each) prior to placement in the storage solutions. The storage solutions were either PBS containing 30% sucrose and 0.1% $NaN_3$ (tissue for cryostat sectioning) or PBS containing 0.1% $NaN_3$ (tissues for wholemount preparation). Sections were cut at 8–14 $\mu$m and then thaw attached to either chrome alum-gelatin or polyornithine coated slides, and were then placed under vacuum in a dessiccator containing phosphorous pentoxide for 30 min, prior to incubation with the primary antisera.

Tissue specimens were incubated with primary antisera (Table 1) diluted in PBS for 24 to 48 h in a humid chamber at room temperature. Anti-NSE and anti-S100

**Table 1** Primary antisera and dilutions

| *Tissue Antigen* | *Host* | *Raised Against* | *Dilution* | *Dako Code* |
|---|---|---|---|---|
| NSE | Rabbit | Bovine Brain NSE | 1:500 | A589 |
| S100 | Rabbit | Ox Brain S100 | 1:300 | Z311 |
| Desmin | Mouse | Porcine Stomach Desmin | 1:10 | M724 |

were used to locate neural tissue and anti-desmin was used for muscle identification. Horse or human serum (10%) was added to the primary antibody mixture when incubating sectioned tissue to decrease non-specific binding and background fluorescence. The addition of non-immune serum was not necessary for the wholemount preparations.

Following the primary antiserum incubation the specimens were washed with PBS for 15 min prior to the application of the fluorescein isothio cyanate or horseradish peroxidase conjugated secondary antiserum for 1h. The peroxidase reaction was developed with diaminobenzidine. Following the second incubation the specimens were washed with PBS and mounted with buffered glycerol (pH 8.6). The slides were viewed in a Leitz epifluorescence microscope with an attached camera system for photography.

All tissues prepared for bright-field microscopy were fixed and stored in 4% formaldehyde at room temperature. This tissue was dehydrated and embedded in paraffin and sections of 5–10 $\mu$m thickness were cut longitudinally, transversely or obliquely through the sphincter segment or the sphincter-duodenal junction. Longitudinal and transverse sections of the same thickness were cut from the gallbladder and common bile duct. The sections were stained with haemotoxylin and eosin, van Geison, or periodic acid-Schiff reagents.

### TISSUE PREPARATION

### *Gallbladder*

Seventeen gallbladders were prepared for fluorescence immunohistochemical examination in section or wholemount. Specimens for wholemount analysis (9 gallbladders) were pinned on balsa wood under tension to decrease the thickness of the tissue. To prepare the wholemounts of the gallbladder, the stored tissue was placed in a petri dish containing PBS, and the dissection performed with the aid of a dissecting microscope. The epithelium was gently scraped from the mucosal surface with a sharp scalpel. The tissue layers of the gallbladder were then teased apart with watchmakers forceps.

### *Common Bile Duct*

A segment, approximately 2cm long, of the mid-portion of the common bile duct from three possums, was fixed intact. The lumen of the bile duct was cannulated with a fine catheter and perfused with the fixative to ensure complete fixation of the wall. A common bile duct segment from four animals was fixed in 4% formaldehyde for histology.

### *Sphincter of Oddi and Duodenum*

The sphincter segment and adjacent duodenum was removed from 12 possums. Pancreatic tissue was then dissected away from the wall of the sphincter segment, as far as the junction of the pancreatic duct with the common bile duct. Eight sphincter segments were fixed intact; the duodenum was slit longitudinally, opposite the junction with the sphincter (i.e. along the antimesenteric border) and pinned flat on balsa wood, with the sphincter held perpendicular to the duodenum.

In four animals the sphincter segments were opened along the lumen of the common bile duct as far as the duodenal wall, pinned on balsa wood, and fixed in 4% formaldehyde prior to preparation for histology.

### RESULTS

### *Macroanatomy*

Figure 1 is a scale drawing of the organisation of the extrahepatic biliary system in the Australian possum. The gallbladder was attached by a short mesentery to the undersurface of the right lobe of the liver. The duodenum of the possum was suspended on a mesentery from the coeliac and mesenteric arterial axis. Contained within this mesentery were the common bile duct, portal vein and hepatic artery; also enveloped within the mesentery was the pancreas, which surrounded the terminal bile duct and adjacent vascular structures.

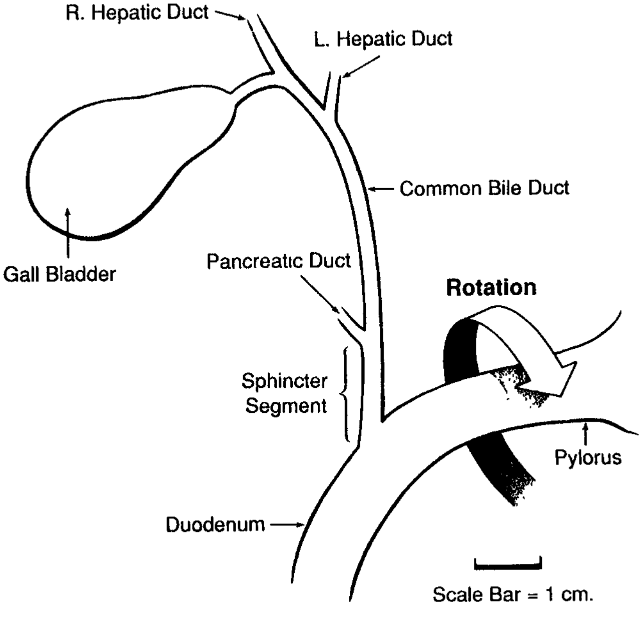

**Figure 1** The macroscopic arrangement of the biliary tree in the Australian possum. The sphincter segment of the common bile duct lies behind (dorsal to) the duodenum: the duodenum has been rotated, as indicated, so that the sphincter region can be depicted. The left hepatic duct has been drawn as a single duct, although multiple ducts from the left hepatic lobules usually join the right duct.

### *Microanatomy*

The arrangement of the tissue layers of the possum gallbladder was identical to that of other mammals. The common bile duct wall consisted mainly of connective tissue and blood vessels, but scattered bundles of longitudinally orientated muscle fibres were occasionally seen, particularly in the distal duct close to the junction with the pancreatic duct.

The structure of the sphincter Oddi and the adjacent duodenum is illustrated diagrammatically in Figure 2. The lumens of the pancreatic duct and the common bile duct ran parallel to each other within the sphincter segment (Figures 2, 3a) and united to form the ampulla within the submucosa of the wall of the duodenum. The resultant common channel was short and entered the duodenal lumen at a point marked by a small papilla (Figure 3b).

The external sphincter muscle layer was a continuation of the muscularis externa of the duodenum. The internal sphincter muscle layer (Figure 3a,c) consisted of an outer circular and an inner longitudinal layer (Figure 3d). The inner longitudinal muscle formed a "figure of eight" in transverse section, in this way surrounding the pancreatic and common bile duct lumens independently, whereas the circular layer of the internal sphincter, and the external sphincter, enclosed both ducts together (Figure 2). Thus, the muscle fibres in the fibromuscular septum between the two

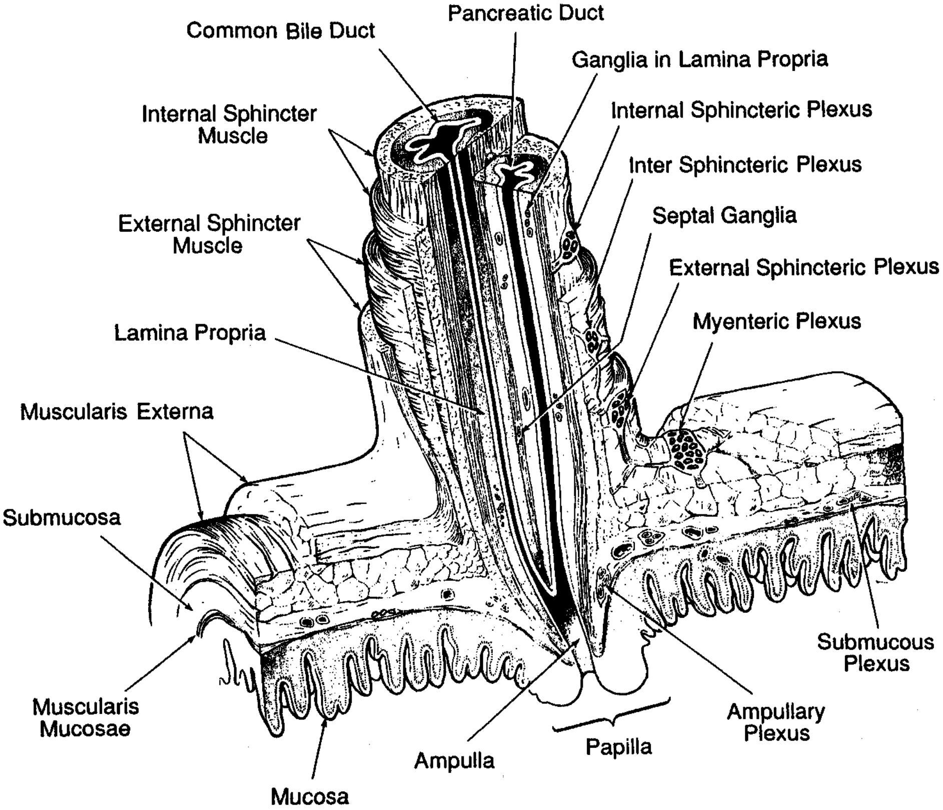

**Figure 2** Diagrammatic representation of the choledochoduodenal junction, reconstructed from analysis of sections. The common bile and pancreatic ducts are mutually enveloped by the sphincter muscle, and their lumens join to form an ampulla within the duodenal wall. Four groups of ganglia are found in the sphincter: three ganglionated plexuses (the external sphincteric plexus, the intersphincteric plexus and the internal sphincteric plexus) and scattered ganglia in the lamina propria and in the septum between the common bile duct and the pancreatic duct.

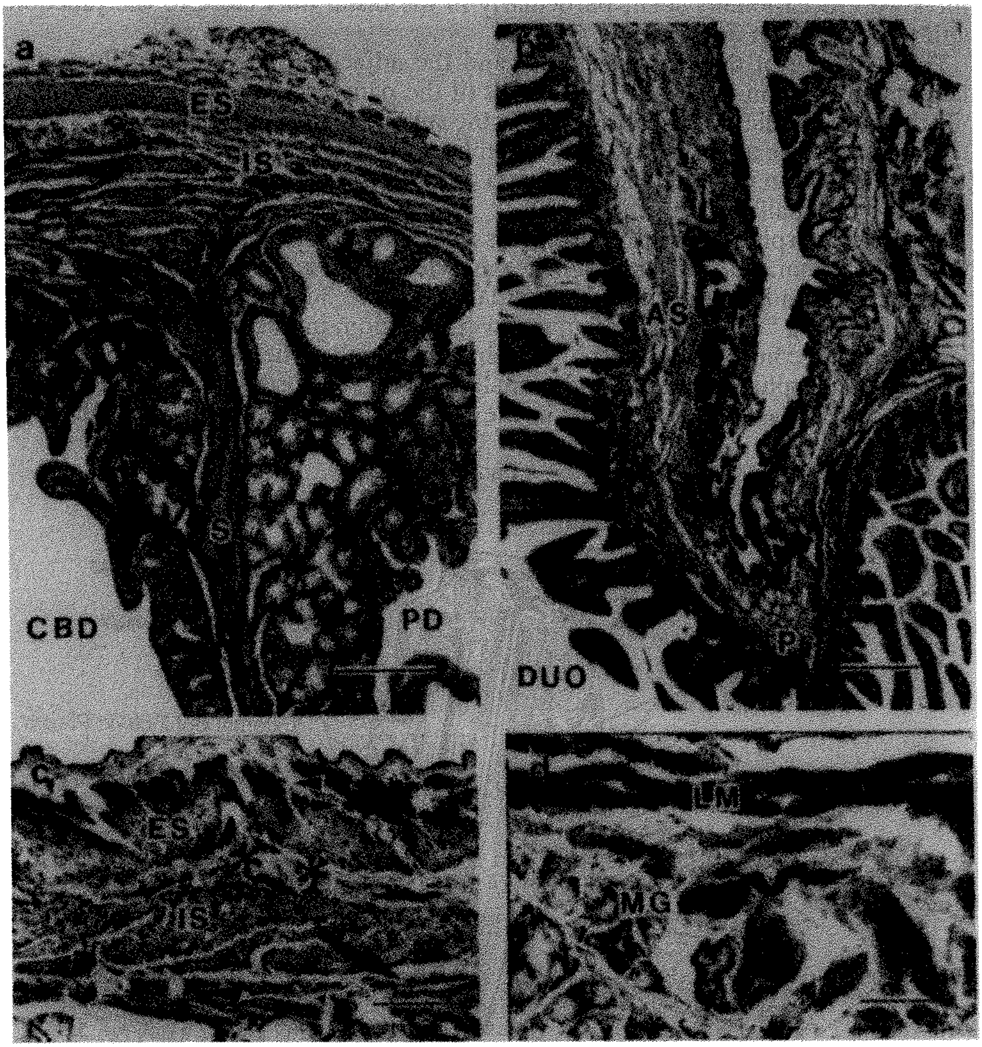

**Figure 3** Micrographs to illustrate the structure of the sphincter of Oddi segment (horseradish peroxidase conjugated antiserum). a. van Geison: transverse section through the extra-duodenal segment of the sphincter. The external sphincter muscle (ES) and the internal sphincter muscle (IS) can be seen in the wall of the sphincter. The septal muscle (S) is located between the common bile duct (CBD) and the pancreatic duct (PD). b. van Geison; longitudinal section of the ampulla of the sphincter (AS). A small papilla (P) is formed at the site of entry of the duct into the duodenum (DUO). The actual entry is out of the plane of this section. c. Longitudinal section of the muscle wall of the sphincter segment, stained for the immunohistochemical localisation of neuron specific enolase. There is some non-specific staining of the smooth muscle, particularly the outer longitudinal fibres. The nerve cell bodies are darkly stained and the nerve fibres bundles are the black dots or strands. The external sphincteric plexus (arrowheads) is on the outer aspect of the circular muscle layer of the external sphincter (ES). The intersphincteric plexus (asterisks) is located between the external sphincter and the internal sphincter (IS). The internal sphincteric plexus (arrow) is on the inner aspect of the internal sphincter. d.Longitudinal section of the sphincter mucosa, with a mucosal ganglion (MG) deep to the inner longitudinal muscle layer (LM). Calibrations: a,100$\mu$m; b,200$\mu$m; c,50$\mu$m; d,25$\mu$m ducts were predominantly longitudinal in orientation.

The muscle layers comprised approximately 50% of the thickness of the sphincter wall and the contributions from the external and the internal sphincters were approximately equal. The muscle extended along the extraduodenal sphincter segment for approximately 9 of its 15mm (measured under the microscope on 3 specimens in which sections were cut longitudinally). The hepatic end of the sphincter segment, from the junction of the pancreatic and common bile ducts for a distance of approximately 6mm, contained only occasional bundles of longitudinal muscle fibres in the connective tissue of the wall.

As the sphincter segment passed obliquely through the duodenal wall, the circular layer of the internal sphincter muscle continued beyond the duodenal muscularis externa and extended into the duodenal submucosa. The innermost longitudinal muscle continued beyond the limit of the circular muscle and was contiguous with the muscularis mucosae of the duodenum (Figure 2).

The lamina propria of both the pancreatic duct and the common bile duct contained many PAS positive, mucin-producing, glands.

### ARRANGEMENT OF NERVE PLEXUSES

### *Gallbladder*

The arrangement of neural plexuses in the gallbladder is shown schematically in Figure 4. The serosa contained a network of large nerve trunks which extended over the wall of the gallbladder. Some of the trunks were located in close relation to

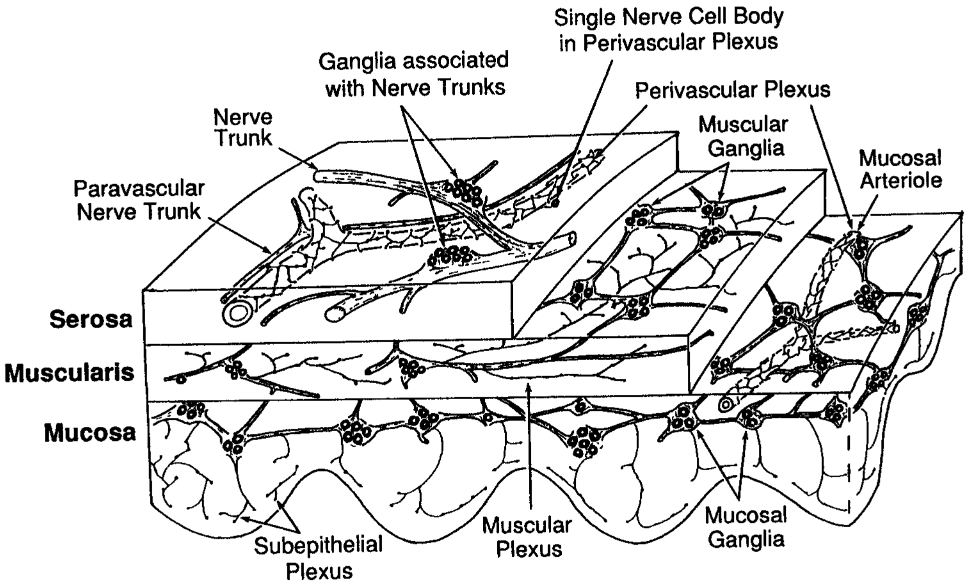

**Figure 4** Diagram to show the arrangement of ganglia and nerve fibres in the wall of the gallbladder.

the blood vessels, but others followed an independent course. Nerve trunks divided and spread throughout the serosa, and their branches penetrated to the muscle layer and mucosa. The nerve trunks, were largest in the serosa near the junction of the cystic duct with the gallbladder and tended to decrease in size as they spread towards the fundus; they further decreased in size as they penetrated the inner layers.

Ganglia were associated with the nerve trunks in the serosa and muscle (Figure 5). Generally, the ganglia were attached to the sides of the trunks (Figure 5c) and contained up to approximately 20 nerve cell bodies; other ganglia were found at junctional points of the nerve trunks (Figure 5b). The sizes of the ganglia (i.e. the number of nerve cell bodies) tended to be directly proportional to the size of the nerve trunk; the largest ganglia being associated with nerve trunks in the serosa near the junction of the cystic duct with the gallbladder. Occasionally, 1 or 2 nerve cell bodies were contained within the trunks. Ganglia, separate from those associated with the large nerve trunks, were found in the muscle and mucosa (Figure 6). These ganglia varied in size from a single nerve cell body to 10 or 11 nerve cell bodies. Within each layer, they were linked by connecting strands of nerve fibres; in the muscle they were also linked to the nerve trunks by connecting strands. Examination of large wholemount preparations indicated that the frequency and size of these ganglia in the muscle and mucosa did not vary between regions of the gallbladder.

Varicose nerve fibres were situated on the outer surface of the muscle bundles and the nerve fibres coursed predominantly in the direction of the muscle fibres. A dense subepithelial plexus was formed by varicose nerve fibres arranged in a network. Varicose nerve fibres could be traced to the subepithelial plexus from nerve cell bodies in the mucosa, and to the muscular plexus from nerve cell bodies in the muscle layer. Possible connections from the ganglia of the muscle layer to nerve fibres in the mucosa or from mucosal ganglia to nerve fibres in the muscle could not be traced because the two layers were examined as separate wholemount preparations. Sectioned specimens were found to be unsuitable for tracing the projections. A perivascular plexus of varicose nerve fibres surrounded arterioles in the serosa, muscle (Figure 6b) and mucosa. A paravascular plexus of small nerve trunks was associated with the larger serosal arteries and nerve fibres frequently connected the para- and perivascular plexuses. Occasionally, small ganglia (1 or 2 nerve cell bodies) were incorporated in the paravascular plexus and fibre projections from these nerve cell bodies joined the perivascular plexus.

### *Common Bile Duct*

The neural architecture of the common bile duct was distinctive in that there were large, longitudinally oriented nerve trunks, both within the wall and lying on the outer surface of the wall (Figure 7a). Ganglia were sparsely distributed and not observed in most sections; when found, the ganglia were usually located in the outer aspect of the wall. There was a dense subepithelial and periglandular plexus of varicose nerve fibres and a perivascular nerve plexus associated with both extramural and intramural arterioles.

### *Sphincter of Oddi and Duodenum*

The ganglionated plexuses of the sphincter of Oddi were arranged in relationship to

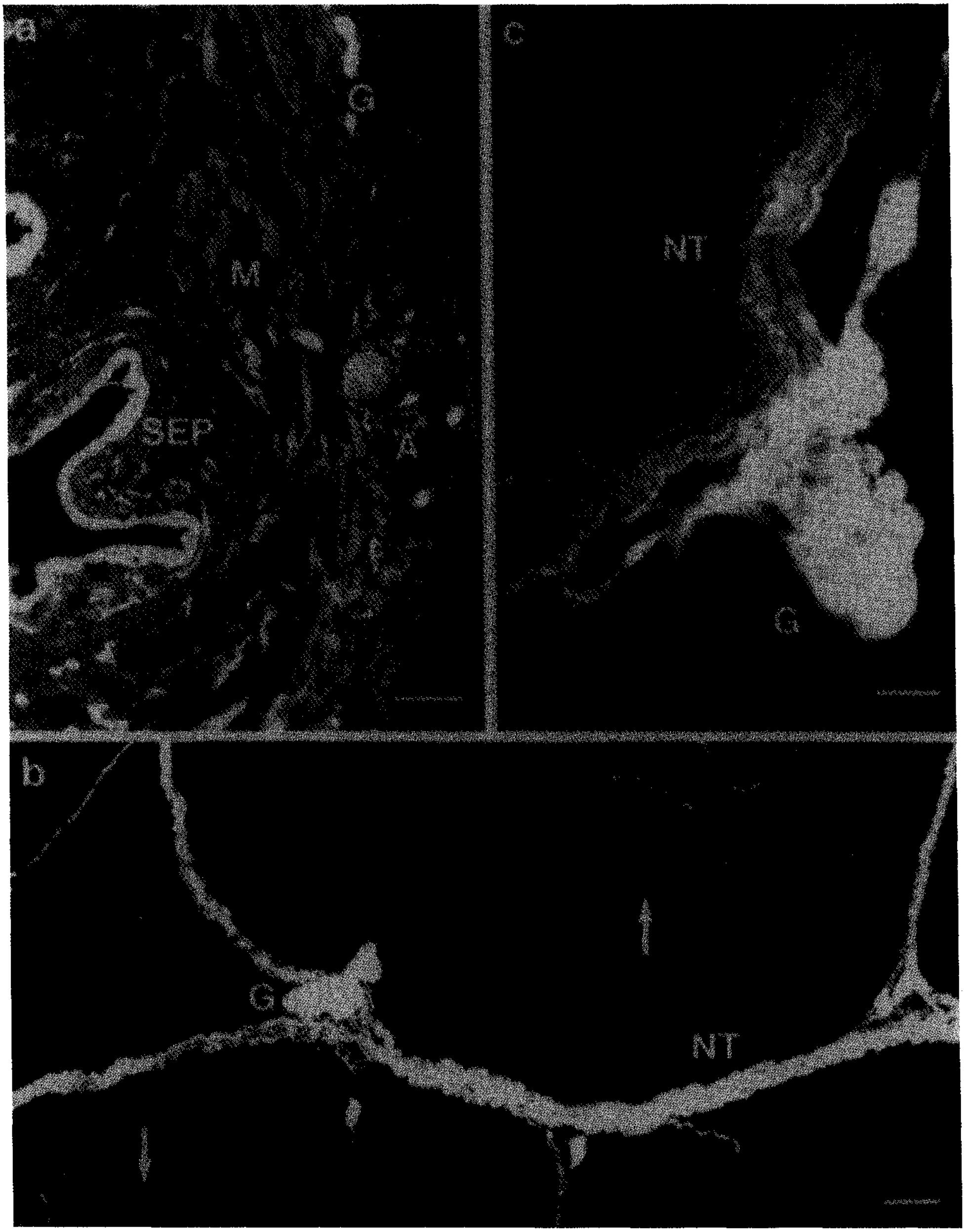

**Figure 5** Immunofluorescence localisation of neuron specific enolase in the gallbladder. a. Section showing ganglia (G) in the serosa and nerve fibres of the muscle layer (M) and in the subepithelial plexus (SEP). The staining of the epithelium appears to be non-specific. b. A montage of the serosa with large nerve trunks (NT) and ganglia (G) at junctional points of the trunks. Small nerve fibre bundles separate from the nerve trunks can be seen (arrows). c. A large nerve trunk (NT) in the serosa with an attached ganglion (G). Calibrations: a,b. 100$\mu$m; c, 50$\mu$m

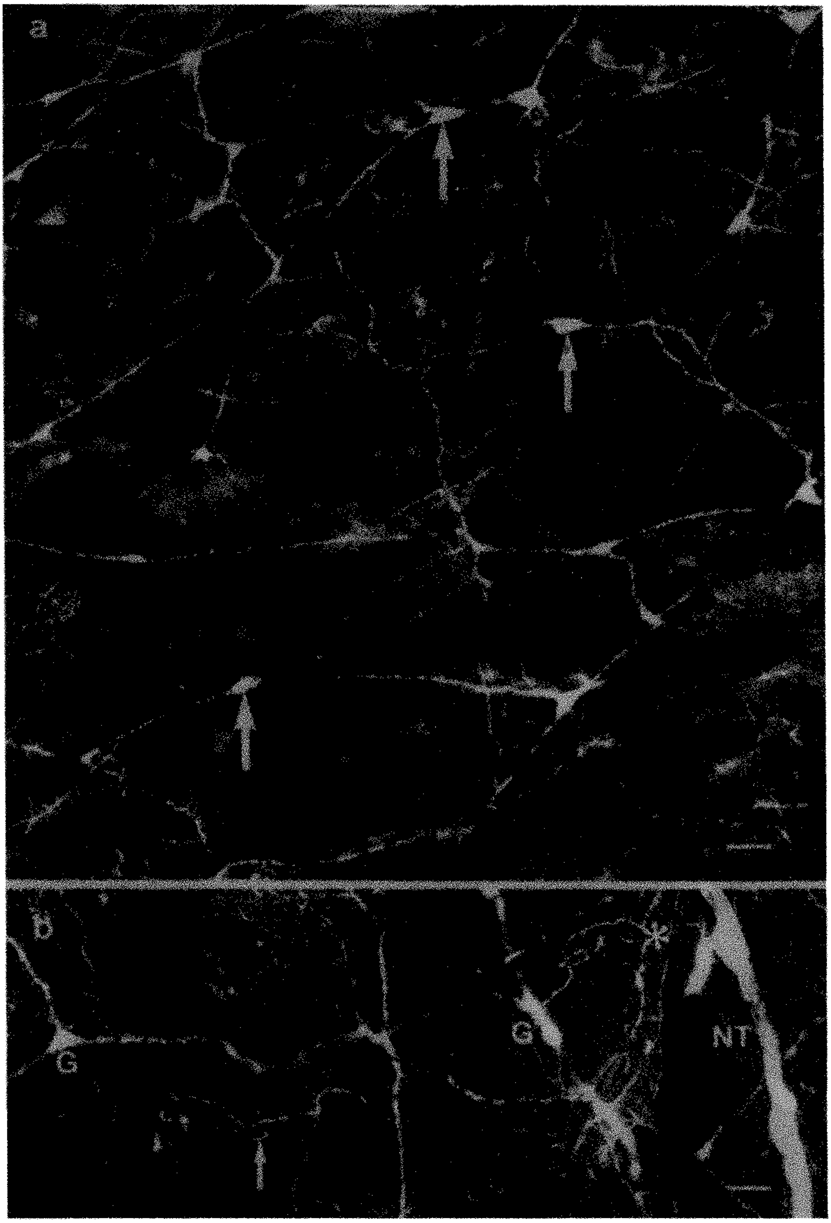

**Figure 6** Immunofluorescence for neuron specific enolase in montages of wholemount preparations of the mucosa (a) and muscle (b) layers of the gallbladder. a. The mucosal ganglionated plexus with ganglia (arrows) and interconnecting nerve strands. Some fine varicose subepithelial plexus nerve fibre bundles can also be seen. b. The muscular plexus component of the outer ganglionated plexus with a large nerve trunk (NT) and some ganglia (G), not attached to the nerve trunk, but linked by connecting fibre bundles. An intramuscular blood vessel is outlined by a perivascular plexus (asterisk) and some fine varicose nerve fibre bundles are seen within the muscle (arrow). Calibrations: a and b, 100$\mu$m the muscle layers as depicted in Figure 2. The external sphincteric plexus was located between the longitudinal and circular muscle layers of the external sphincter muscle (Figure 3c) and was continuous with the myenteric plexus of the duodenum. An intersphincteric ganglionated plexus was found between the external and internal sphincter musculature and an internal sphincteric plexus was located between the circular and longitudinal muscle layers of the internal sphincter. The position of the ganglia in the latter plexus was less well defined as ganglia were often intermingled with the longitudinal muscle fibres. Ganglia were situated in the fibromuscular septum between the two ducts, once again interspersed among the muscle fibres (Figure 7d). It appeared that the internal sphincteric plexus was continuous with the plexus of septal ganglia. Less frequently, ganglia were present in the lamina propria (Figure 3d) usually close to the inner aspect of the longitudinal muscle. This arrangement of the ganglionated plexuses in the hepatic aspect of the sphincter ceased at the region of termination of the muscle layers.

At the duodenal end of the sphincter, in the region of termination of the inner circular muscle, the ganglia of the intersphincteric plexus and the internal sphincteric plexus were no longer separated. Furthermore, the duodenal submucous plexus ganglia and the ganglia of the inter- and internal sphincteric plexuses were closely related as the sphincter passed through the submucosal portion of the duodenal wall. The ganglionated network in this region has been called the ampullary plexus. It is probable that the inter- and internal sphincteric plexuses are continuous with the duodenal submucous plexus, via the ampullary plexus. However, it was not possible to determine whether direct connections existed by the methods used in this study.

Nerve trunks, in association with the mesenteric blood vessels, were located at the external surface of the sphincter wall. Intramural nerve fibre bundles were found in the ganglionated plexuses and around arterioles. With the exception of the outer longitudinal muscle, the muscle layers had a rich distribution of varicose nerve fibres, generally orientated in the direction of the muscle fibres. In addition, the glands in the lamina propria were surrounded by networks of varicose fibres and a plexus of varicose nerve fibres was situated adjacent to the epithelial cells of both the pancreatic and common bile ducts (Figures 5a and 7a).

### DISCUSSION

The choledochoduodenal junction in all species reported has a muscular wall. However, the arrangement of the muscle layers, and the contribution of the duodenal musculature to the sphincter segment, varies significantly amongst species. Furthermore, the method of termination of the common bile duct and pancreatic duct, and the presence or absence of an ampulla of Vater, varies between species[4].

The American opossum[5] and the Australian possum have a substantial length of sphincter that is extra-duodenal. However, there is one major difference between these species. The lumens of the common bile duct and the pancreatic duct of the American opossum unite to form an ampulla, prior to the junction of the sphincter segment with the duodenum, an arrangement quite different to that in the Australian possum, in which the ampulla is formed within the duodenal wall. In all

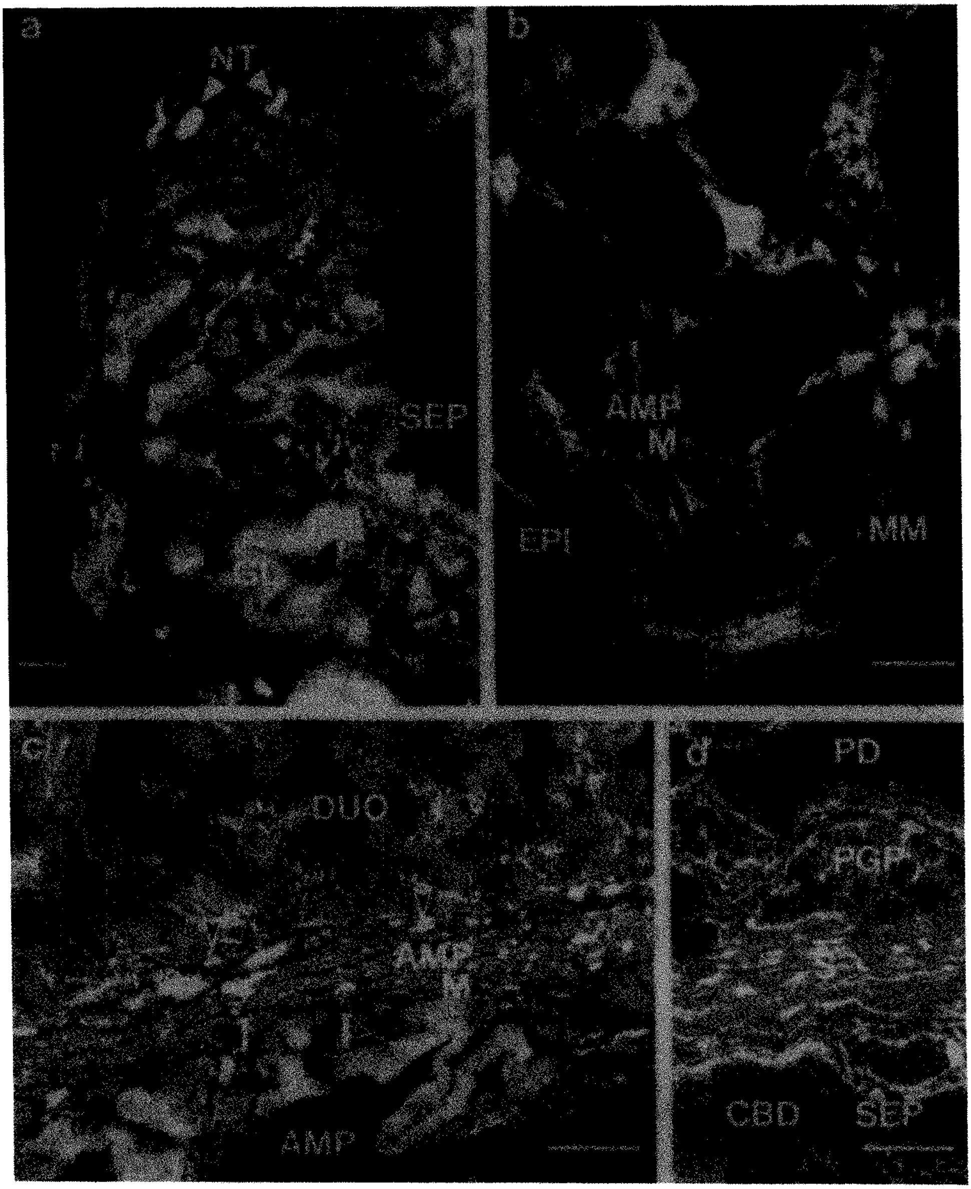

**Figure 7** Sections showing nerve fibre bundles and structural features of the common bile duct and the sphincter of Oddi. a. Transverse section of the common bile duct showing nerve fibre bundles in relation to an artery (A) intramural glands (GL) and in the subepithelial plexus (SEP). Nerve trunks (NT) are found within the wall of the duct. b. A section of the terminal sphincter segment incubated with anti-S100 antiserum. The continuity between the terminal ampullary muscle of the sphincter (AMP M) and the muscularis mucosae of the duodenum (MM) is demonstrated. Reactive nerve fibres are in the sphincter muscle. The epithelium of the sphincter (EPI), adjacent to the junction with the lumen of the duodenum, is also seen. c. Immunofluorescence for neuron specific enolase in a longitudinal section of the choledochoduodenal junction in which both the lumen of the duodenum (DUO) and the lumen of the ampulla (AMP) can be seen. There are nerve cell bodies in the ampullary muscle (arrows) and nerve cell bodies in the submucous plexus of the duodenum (arrowheads). Nerve fibre bundles are seen as white dots or lines. d. Immunofluorescence for neuron specific enolase in a longitudinal section of the sphincter septum (S) which separates the pancreatic duct (PD) and the common bile duct (CBD). Numerous nerve fibre bundles are present in the subepithelial plexus (SEP) the periglandular plexus (PGP) and between the muscle bundles in the septum. Calibrations: a,100$\mu$m; b,50$\mu$m; c and d 100$\mu$m other mammalian species examined, i.e. human, cat, dog, guinea-pig, sheep, goat and chimpanzee, the extraduodenal component of the sphincter muscle is either small or absent[4,6,7,8,9,10]. The anatomy of the human sphincter of Oddi has been extensively studied by Boyden[7]. He considered the human sphincter to be independent of duodenal musculature, and made the observation that it developed five weeks later than the duodenum. The human sphincteric muscle is primarily intraduodenal with a small, but variable, extraduodenal component of circular muscle (the superior choledochal sphincter).

The gallbladder of all species examined contains plexuses of nerve fibres and ganglia, which in general morphology resemble the submucous plexus of the small intestine[11,12,13]. This is also true of the Australian possum. A variety of techniques have been applied to the guinea-pig gallbladder[11,12,13], which contains a single ganglionated plexus adjacent to the outer aspect of the muscle layer. In contrast to the Australian possum, the neural plexus within the muscle layer of the guinea-pig gallbladder is not ganglionated. Sutherland[11] found some nerve cell bodies within the mucosa of the guinea-pig gallbladder, but their existence was not confirmed by the other investigators.

In the gallblader of all other species examined (cats[14,15,16], dogs[16,17], monkeys[11,15], and humans[14,18,19,20]) nerve cell bodies are found within the muscularis and mucosa. The human gallbladder, like the Australian possum, has three ganglionated plexuses; a serosal, myenteric, and mucosal. Large nerve trunks in the serosa are associated with large ganglia at the junctional points and the mucosal ganglia are generally only single nerve cell bodies. The subepithelial plexus of nerve fibres is formed by neural projections from both the myenteric and mucosal ganglia[19].

Although the ganglionated plexus of the gallbladder and the submucous plexus of the small intestine have features in common (e.g., plexus morphology and the number of nerve cell bodies per ganglion [12,13]) differences also exist. Individual nerve cells in the gallbladder are larger[12,13], have a simpler branching pattern, a lesser degree of innervation and a greater glial investment[21] and different neural chemistry[22] when compared to submucous plexus nerve cells. Therefore, it is probably appropriate to consider the nervous system of the gallbladder as a separate and specialised component of the enteric nervous system. Furthermore, the existence of intrinsic ganglia in the wall of the gallbladder, and the location of varicose nerve fibre bundles in the muscle and mucosa, suggests that neuronal mechanisms may have an important role in modulating gallbladder function.

The neural architecture of the common bile duct has not been extensively investigated, partly because of the difficulty in making wholemount preparations. Generally, the findings in the species examined (human[14,19], guinea-pig[12], cat[14,16] and dog[16]) are consistent with those in the Australian possum. Distinctive are the large nerve trunks at the outer aspect of the wall and it is likely that these trunks contain the nerve fibres projecting from nerve cell bodies in the duodenum (and possibly the sphincter of Oddi) to the gallbladder[13,23]. Bile duct ganglia tend to be smaller, and less numerous than gallbladder ganglia (except in the guinea-pig[12]), but supply a rich innervation to the glands and epithelium.

There is less agreement on the innervation of the choledochoduodenal junction. Kyosola[8] observed a rich distribution of nerve fibres and cells in the sphincter region of the cat and dog, but concluded that many of the nerve fibres in the sphincter muscle were derived from neurons in the submucous or myenteric plexuses of the duodenum, rather than from intrinsic sphincter ganglia. Nerve fibres were not found in the mucosa of the dog sphincter. This region in the feline sphincter contained small ganglia and nerve fibres, but no fibres adjacent to the epithelium. In contrast, in the present study of the Australian possum, a dense subepithelial plexus of varicose nerve fibres was found in the sphincteric mucosa. Furthermore, the presence of ganglia in the muscle layers and the mucosa suggests that many of the nerve fibres in the muscle and mucosa were projections of intrinsic nerve cells.

The nerve supply of the cat choledochoduodenal junction had previously been examined by Schulze and Boyden[24]. They described a ring of "transitional" ganglia, located between the duodenal myenteric plexus ganglia and intrinsic ganglia of the sphincter. Kyosola[8] observed a ring of "fairly large and conspicuous ganglia" in the cat choledochoduodenal junction, associated with the duodenal submucous plexus. In the present study, the ganglia of the submucous plexus of the duodenum and the ganglia of the inter- and internal sphincteric plexuses were in close relation, but there was not a change in size of ganglia or aparent transition between the two regions. There was also no apparent change in morphology of the ganglia in the region of continuity between the myenteric plexus of the duodenum and the external sphincteric plexus.

Studies of the innervation of the sphincter segment of the human have been hampered by the lack of availability of suitable specimens. However, it contains a well developed intrinsic plexus, with numerous ganglia, which may be a continuation of the myenteric plexus of the duodenum[19].

Thus, in most studies examining the innervation of the choledochoduodenal junction, there is general agreement that a rich distribution of nerves and ganglia exist in the muscle and mucosa of the sphincteric segment. The projections of neurons in the ganglionated plexuses of the sphincteric segment, and their relationship to the duodenal ganglionated plexuses, remain unclear.

### *Acknowledgements*

This work was supported by grants from the Royal Australasian College of Surgeons and the National Health and Medical Research Council of Australia. Dennis Jones is thanked for his assistance with the figures. The use of Australian brush tailed possums (Trichosurus vulpecula) for all procedures in this study was approved by the Animal Welfare Committee of the Flinders University of South Australia (approval number 188/85) and the National Parks and Wildlife Service Division of the Environment and Planning Department of the South Australian Government (permit number Y12712).

*(Accepted by S. Bengmark 10 March 1993)*

### *References*

1. Iannos, J., Saccone, G.T.P., Bushel, M., Baker, R.A. and Toouli, J. (1989) Gallbladder and sphincter of Oddi response to cholecystokinin in the Australian possum. *J. Gastroenterol. Hepatol.*, **4**, 497–503
2. Calabuig, R., Saccone, G.T.P. and Toouli, J. (1989) Inhibition of sphincter of Oddi motility in the Australian possum by cholic acid. *Surgery*, **106**, 872–878
3. Baker, R.A., Saccone, G.T.P. and Toouli, J. (1990) Cisapride inhibits motility of the sphincter of Oddi in the Australian possum. *Dig. Dis. Sci.*, **35**, 711–715

4. Boyden, E.A. (1937) The sphincter of Oddi in man and certain representative mammals. *Surgery*, **1**, 25–37
5. Dubois, F.S. and Hunt, E.A. (1932) A comparative study of the emptying of the gallbladder in the opossum and the cat together with notes on the anatomy of the biliary tract of the opossum. *Anat. Rec.*, **54**, 289–306
6. Boyden, E.A. (1955) The choledocho and pancreaticoduodenal junctions in the chimpanzee. *Surgery*, **37**, 918–927
7. Boyden, E.A. (1957) the choledochoduodenal junction in the cat. *Surgery*, **41**, 773–786
8. Kyosola, K. (1976) Structure and innervation of the choledochoduodenal junction. *Ann. Chir. Gynaecol.*, **65** (suppl) 192, 1–135
9. Cai, W.Q. and Gabella, G. (1983) The musculature of the gall-bladder and biliary pathways in the guinea-pig. *J. Anat.*, **136**, 237–250
10. Abdalla, O. and Sack, W.O. (1985) The choledochoduodenal junction in sheep and goat. *Zbl. Vet. Med. C. Anat. Histol. Embryol.*,, **14**, 6–14
11. Sutherland, S.D. (1966) The intrinsic innervation of the gallbladder in Macaca rhesus and Cavia porcellus. *J. Anat.*, **100**, 261–268
12. Cai, W.Q. and Gabella, G. (1983) Innervation of the gallbladder and biliary pathways in the guinea-pig. *J. Anat.*, **136**, 97–109
13. Mawe, G.W. and Gershon, M.D. (1989) Structure, afferent innervation, and transmitter content of ganglia of the guinea-pig gallbladder: relationship to the enteric nervous system. *J. Comp. Neurol.*, **283**, 374–390
14. Alexander, W.F. (1940) The innervation of the biliary system. *J. Comp. Neurol.*, **72**, 357–370
15. Sutherland, S.D. (1967) The neurons of the gallbladder and gut. *J. Anat.*, **101**, 701–709
16. Kyosola, K. (1978) Adrenergic and cholinergic innervation of the supraduodenal common bile duct. *Am. J. Gastroenterol.*, **70**, 179–183
17. Keast, J.R., Furness, J.B. and Costa, M. (1985) Distribution of certain peptide-containing nerve fibres and endocrine cells in the gastrointestinal mucosa in five mammalian species. *J. Comp. Neurol.*, **236**, 413–422
18. Inoue, H. (1955) A historical study of sensory nerves in the biliary tract. *Arch. Jap. Chir.*, **24**, 257–268
19. Burnett, W., Gairns, F.W. and Bacsich, P. (1964) Some observations on the innervation of the extrahepatic biliary system in man. *Ann. Surg.*, **159**, 8–26
20. Kishimoto, S., Okamoto, K. Shimizu, S., Kambara, A., Kawanishi, M. Yamamato, M. Iwasaki, Y. Kajiyama, G., Miyoshi, A. and Yanaihara, N. (1984) VIP-ergic innervation of the gallbladder in health and disease. *Hiroshima J. Med. Sci.*, **33**, 185–191
21. Cornbrooks, E.B., Pouliot, W.A. and Mawe, G.M. (1992) Structure of neurons and ganglia of the guinea pig gallbladder: light and electron microscopic studies. *J. Comp.Neurol.*, **317**, 31–44
22. Talmage, E.K., Pouliot, W.A., Cornbrooks, E.B. and Mawe, G.M. (1992) Transmitter diversity in ganglion cells of the guinea pig gallbladder: an immunhistochemical study. *J. Comp. Neurol.*, **317**, 45–56
23. Padbury, R.T.A, Furness, J.B., Baker, R.A., Toouli, J. and Messenger, J.P. (1993) Projections of nerve cells from the duodenum to the sphincter of Oddi and gallbladder of the Australian possum. *Gastroenterology* **104**, 130–136
24. Schulze, J.W. and Boyden, E.A. (1943) The blood supply and innervation of the choledochoduodenal junction in the cat. *Anat. Rec.*, **86**, 15–39

*(Accepted by S. Bengmark 10 March 1993)*

### INVITED COMMENTARY

Padbury, Baker, Messenger, Toouli, and Furness have provided an exquisite description of neural net that modulates the motor function of the gallbladder and biliary sphincter of the Australian bush-tailed possum. They have clearly demonstrated the anatomic differences between this "critter" and the opossum extensively utilized for the study of biliary function in our laboratory. The advantage of the latter relates to the fact that the pancreatic duct joins the lower end of the bile duct outside of the duodenal wall thereby providing a long common channel for experimental manipulation. Senninger, working in our laboratory, took advantage of this arrangement to study the role of bile reflux in the pathogenesis of acute necrotizing pancreatitis. Calabuig, who has worked with both Australian possum and the American opossum has taught me that each species has a uniqueness that is invaluable to unravelling the mysteries of biliary function. The possum is better suited for studying normal physiologic processes. The opossum is ideal for electromyographic measurements and experiments that require instrumentation, since they are larger and appear more docile than the possum. A major difference, however, likely relates to how these species have evolved. The opossum's biliary sphincter is a pump, whereas it is likely that the possum's sphincter is a resistor such as is the case in the cat. Why the difference? My explanation (speculation) is that the oposum would forage for food at night and digests its nutrients during the day, protected from its predators by hanging high in the branch of a tree by its prehensile tail. This provides a logical explanation for the biliary sphincter serving as a pump to deliver bile into the duodenum against the force of gravity. It now remains for someone to define the neural nets of the opossum in order to compare the differences in "wiring" to those functions.

Frank Moody

Houston, USA

